# Principles and Practice of Surgery

**Published:** 1848-05

**Authors:** 


*Principles and Practice of Surgery.* By the late Geo. McClellan, M. D. 8vo. pp. 432. Grigg, Elliott & Co. Philadelphia: 1848.

The present work was begun, and a portion of the volume had actually gone through the press, at the time of the author's decease, and the task of its completion consequently devolved upon his son, as the rightful guardian of his father's reputation and inheritor of his fame. In the prosecution of his work, the editor has displayed a fidelity in full accordance with filial duty. "Suddenly cut off in the midst of these labours," (alluding to the author's preparation of the volume,) "the whole manuscript was left in much confusion, and many of the subjects in its latter part were incomplete. With little clue to his intentions, other than the knowledge of his opinions, derived from some years of daily practice and observation with him, I have endeavoured to complete all that was left unfinished, in strict accordance with his peculiar views."

The brief space of 432 pages cannot of course be expected to contain anything like a full account either of the principles or practice of surgery; but this deficiency the editor hopes to remedy at some future day, particularly so far as to embrace the author's more important operations. "As now published, the chapters on the different subjects treated of are complete in themselves, and contain most of the important principles of surgery."

